# Echocardiography of the normal camel (*Camelus dromedaries*) heart: technique and cardiac dimensions

**DOI:** 10.1186/1746-6148-8-130

**Published:** 2012-08-03

**Authors:** Mohamed Tharwat, Fahd Al-Sobayil, Ahmed Ali, Sébastien Buczinski

**Affiliations:** 1 Department of Veterinary Medicine, College of Agriculture and Veterinary Medicine, Qassim University, Riyadh, Saudi Arabia; 2 Bovine Ambulatory Clinic, Departement des Sciences Cliniques, Faculté de Médecine Vétérinaire, Université de Montréal, Saint-Hyacinthe, Canada; 3 Permanent address is Department of Animal Medicine, Faculty of Veterinary Medicine, Zagazig University, Zagazig, Egypt

## Abstract

**Background:**

Echocardiography and intra-cardiac dimensions have not previously been reported in adult camels despite its potential application for medical purpose. The aim of this study was to describe the results of a prospective study, aiming to report normal cardiac appearance and normal chamber dimensions in adult camels (*Camelus dromedarius)*.

**Results:**

On the right side, when the probe was placed in the 5^th^ or 4^th^ intercostal space (ICS), the caudal long-axis four-chamber view of the ventricles, atria, and the interventricular septum was obtained. Placing the probe slightly more cranially in the 4^th^ ICS, the caudal long-axis four-chamber view and the caudal long-axis view of the left ventricular outflow tract (LVOT) were imaged. In 7 camels, a hybrid view between a “four-chamber” and “LVOT view” was imaged from the same position. The short-axis view of the ventricles was obtained in the 4^th^ ICS where the transducer was rotated between 0° and 25°. Placement of the transducer in the 3^rd^ ICS allowed visualisation of the right ventricular outflow tract (RVOT). On the left side, when the probe was placed in the 5^th^ or 4^th^ ICS, a four-chamber view was obtained. The LVOT is imaged in the 4^th^ ICS and the RVOT was seen from the 3^rd^ ICS.

**Conclusions:**

This study showed that it is possible to obtain good-quality echocardiograms in adult camels and provide normal cardiac dimensions. This study could be used as a reference for further studies concerning camels with cardiac diseases.

## Background

The dromedary camel is important species in Africa, Middle-East and Australia. Despite their valuable use as draft animals, it is also used for milk and meat production. In the northern hemisphere, it is mainly kept as a companion animal or found in the circus or zoological collections, and can therefore have a worldwide veterinary importance due to the relatively high value [1]. In the camel, heart diseases include pericarditis, vegetative valvular endocarditis, hypertrophic cardiomyopathy, necrotic myocarditis and congenital defects including septal defects, patent ductus arteriosus, transposition of the aorta and pulmonary artery, persistent aortic trunk, and persistent right aortic arch and sarcocystosis [1-7].

The heart diseases listed above are mostly diagnosed at slaughterhouses or incidentally discovered at postmortem examination [3], showing that the diagnosis of camel heart disease is a challenging task especially when typical clinical signs of heart failure are absent. For these reasons, ancillary tests are required to confirm the diagnosis in the living animal. This is of particular importance to avoid further therapeutic investments in a low-value patient when the prognosis is perceived to be poor or to early initiate a treatment in valuable animals.

Echocardiography is a good ancillary tool to assess the heart in other ruminant species. It is a non-invasive, straightforward method for assessment of the bovine heart [8]. It has been used extensively in cattle and can be used as a prognostic tool in some diseases since the extension and importance of the disease are better assessed [9,10]. The procedure is a widely used imaging tool in small animals, horses and cattle for evaluation of morphologic changes, abnormal wall thickness, chamber size and valvular appearance and function [11-13].

In camel practice, ultrasonography has been used mainly for examination of the reproductive tract [14-16]. The data on the medical and surgical use in the camel are rare. To the authors' knowledge, echocardiography of the normal camel heart and its internal dimensions has not previously been reported in the literature. The present study was therefore designed to report normal cardiac chamber appearance and quantitative dimensions in adult camels based on techniques adapted from echocardiographic studies in cattle [8,9,17].

## Methods

### Animals, history and physical examination

Twenty-two adult camels (*Camelus dromedarius*) (age: 8.8 ± 3.8 years) were examined at the Veterinary Teaching Hospital, Qassim University, Saudi Arabia. Of the 22 camels, 2 (9%) were males and 5 (25%) were 2–3 month pregnant females, whilst the others were non-pregnant and non-lactating females. Their BCS ranged from 3.0 to 3.75 and weighed 514 ± 69 kg. All camels received a full clinical examination [18] including general behaviour and condition, auscultation of the heart, lungs, rumen and intestines, detection of heart and respiratory rates and rectal temperature. Camels were clinically healthy based on physical and laboratory evaluation (normal complete cell blood counts and biochemistry panel), and they had full access to feed and water before and after examination. Based on a 1 (very thin) to 5 (fat) scale, the body condition score (BCS) of camels was determined as previously reported [19]. All camels were maintained in free-stall barns under the *Laboratory Animal Control Guidelines* of Qassim University, which basically conform to the *Guide for the Care and Use of Laboratory Animals* of the National Institutes of Health in the USA (NIH publication No. 86–23, revised 1996). The experimental protocol has been approved by the Animal Ethical Committee, Deanship for Scientific Research, Qassim University.

### Echocardiographic protocol

First, the forelimb of each camel was bent and tied with a rope on the carpal joint. The head was then held and the animal was pushed until it was positioned in a sternal recumbency. The fore-and-hind limbs were then tied by a rope near the carpal and hock joints, respectively. All echocardiographic examinations were performed in the recumbent animal. Camels were sedated using xylazine (0.02 mg/kg IV) (Bomazine 10%, Bomac Laboratories Ltd, New Zealand). Echocardiographic examinations were performed using an ultrasound machine with a 3.5 MHz sector transducer (SSD-500, Aloka, Tokyo, Japan). In preparation for the echocardiography, the intercostal spaces (3^rd^ to 6^th^) on both sides of the thorax were clipped, shaved and swabbed with alcohol to remove excess oil, and coupling gel was applied. The third, fourth and fifth intercostal spaces in the cardiac region were examined ultrasonographically on the right and then the left sides of the thorax. The thoracic limbs were moved cranially to facilitate better contact between the probe and the intercostal space. In the cardiac area, the heart, valves and major blood vessels were imaged.

**Figure 1:**
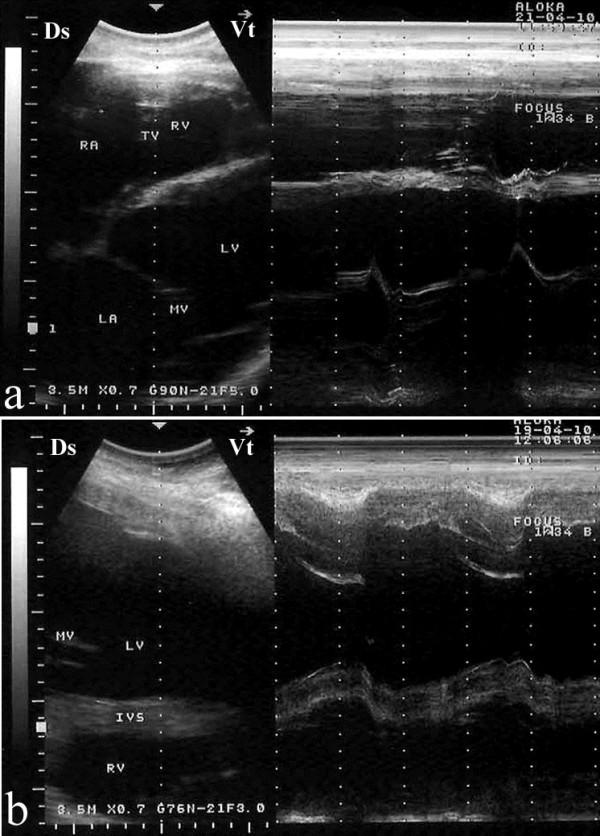
**B-M mode images obtained from both the right (a) and left (b) hemithorax.** Ds, dorsal; Vt, ventral; IVS, interventricular septum; LA, left atrium; LV, left ventricle; MV, mitral valve; RA, right atrium; RV, right ventricle; TV, tricuspid valveB-M mode images obtained from both the right (**a**) and left (**b**) hemithorax. Ds, dorsal; Vt, ventral; IVS, interventricular septum; LA, left atrium; LV, left ventricle; MV, mitral valve; RA, right atrium; RV, right ventricle; TV, tricuspid valve.

The cardiac views obtained in this study are adaptation of those described for cattle [8,9,17]. Four two-dimensional (2-D) parasternal images were obtained from the right and three 2-D parasternal images from the left as described for cattle [10]. sly, M-mode images were obtained from the right and left sides of the thorax. 2-D images from both the right and left hemithorax were used to guide the placement of the probe and obtain accurate M-mode recordings (Figure 1). Coupling gel was applied to the transducer, and this was applied to the skin approximately in the 3^rd^ and 4^th^ and 5^th^right and left intercostal spaces. On the right side, the images were obtained in the following order: a caudal long-axis view of the right and left ventricles, a caudal long-axis view of the left ventricular outflow tract (LVOT), a caudal short-axis view of the ventricles and a cranial long-axis view of the right ventricular outflow tract (RVOT). On the left side, a caudal long-axis view of the heart (four-chamber view), a caudal long-axis view of the LVOT and a cranial long axis-view of the RVOT were obtained. The intercostal space and probe orientation used to obtain each image was recorded at the end of each examination.

Echocardiographic measurements were performed using the electronic ultrasound calipers. Echocardiographic examinations were performed in the camels by the same operator (M.T.). Three non-consecutive cardiac cycles were measured and later measurements were averaged in order to eliminate some of the measurement errors. Eighteen measurements were recorded from the 2-D images. Right ventricular diameter in systole (RVs) and diastole (RVd), right atrial diameter in systole (RAs) and diastole (RAd), right ventricular wall thickness in systole (RVWs) and diastole (RVWd), interventricular septal thickness in systole (IVSs) and diastole (IVSd) and tricuspid valve diameter in systole (TVDs) were measured from the right parasternal caudal long-axis four-chamber view with the probe placed in the 5^th^ intercostal space (ICS) with a slight clockwise rotation or perpendicular in the 4^th^ ICS. Left ventricular diameter in systole (LVs) diastole (LVd), left atrium diameter in systole (LAs) and diastole (LAd), left ventricular wall thickness in systole (LVWs) and diastole (LVWd) and mitral valve diameter in systole (MVDs) were measured from the left parasternal view with the transducer positioned in the 5^th^ or 4^th^ ICS and directed slightly caudodorsally. Aortic diameter in diastole (AOd) was measured from the left parasternal view with the transducer placed in the 4^th^ ICS turned slightly more cranially and rotated slightly counter clockwise. Pulmonary artery diameter in diastole (PAd) was measured from the left parasternal view with the transducer placed obliquely in the 3^rd^ ICS. Systolic measurements were measured during closure of the atrioventricular valves and opening of the semilunar valves, whilst diastolic measurements were measured during opening of the atrioventricular valves and closure of the semilunar valves. Ventricular measurements were measured at the level of the papillary muscles close to the chordae tendinae, whilst atrial measurements were measured at the widest part of the atria. Data are expressed as mean ± SD. At the end of the study, all the camels were slaughtered and their hearts were examined macroscopically.

## Results

The camels in this study had no history or evidence of cardiac dysfunction. No abnormalities were detected on clinical examination of the cardiothoracic systems of any of the animal and no murmurs or dysrhythmia were detected by cardiac auscultation. On clinical evaluation, mean rectal temperature was 36.6 ±0.6 °C (reference range; 36.0-38.0 °C), mean heart rate was 36 ± 8 bpm (reference range; 28–40 bpm) and mean respiratory rate of 10 ± 4 bpm (reference range; 5–12 bpm). Postmortem examination of the camels’ hearts revealed no epicardial, myocardial or endocardial anomalies.

### Right parasternal ultrasonograms

**Figure 2:**
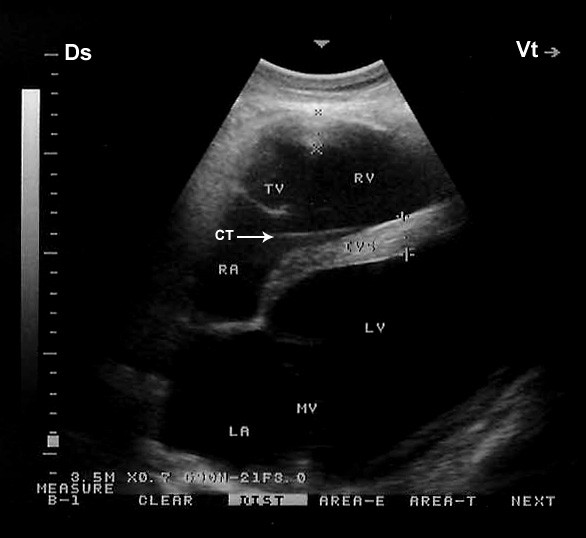
**Right parasternal caudal long-axis view of the left and right ventricles (four-chamber view).** The chordae tendinae of the tricuspid valve are also seen as echoic lines (arrow). Ds, dorsal; Vt, ventral; IVS, interventricular septum; LA, left atrium; LV, left ventricle; MV, mitral valve; RA, right atrium; RV, right ventricle; TV, tricuspid valve; CT, chordae tendinae.

When the probe was placed longitudinally in the 5^th^ intercostal space with a slight clockwise rotation or perpendicular in the 4^th^ ICS, the caudal long-axis four-chamber view of the ventricles, atria, and the interventricular septum was imaged (Figure 2). In this position, the right and left ventricles were visible in all the camels, the tricuspid valve in 20 and the mitral valve and the right and left atria in 19 camels.

**Figure 3:**
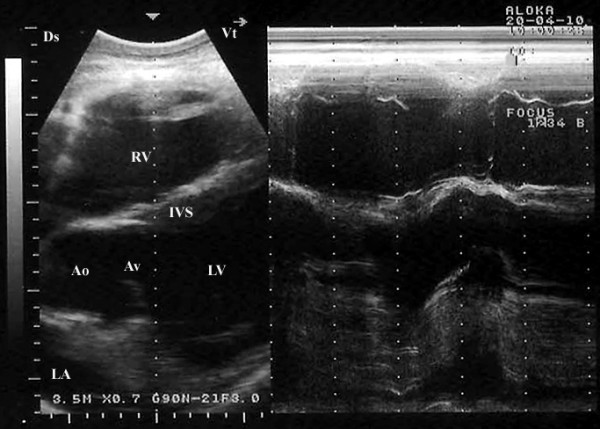
**Right parasternal caudal long-axis view of the left ventricular outflow tract showing both ventricles and interventricular septum together with the aorta and the aortic valve.** Ds, dorsal; Vt, ventral; Ao, aorta; Av, aortic valve; IVS, interventricular septum; LA, left atrium; LV, left ventricle; RV, right ventricle.

**Figure 4:**
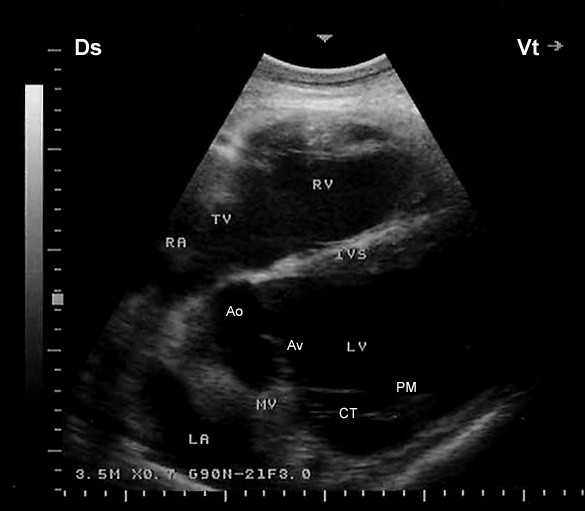
**Right parasternal caudal long-axis view showing the four-chamber view together with the left ventricular outflow tract view.** The chordae tendinae of the mitral valve are also seen as echoic lines Ds, dorsal; Vt, ventral; Ao, aorta; Av, aortic valve; IVS, interventricular septum; LA, left atrium; LV, left ventricle; MV, mitral valve; RA, right atrium; RV, right ventricle; TV, tricuspid valve; CT, chordae tendinae; PM, papillary muscles.

**Figure 5:**
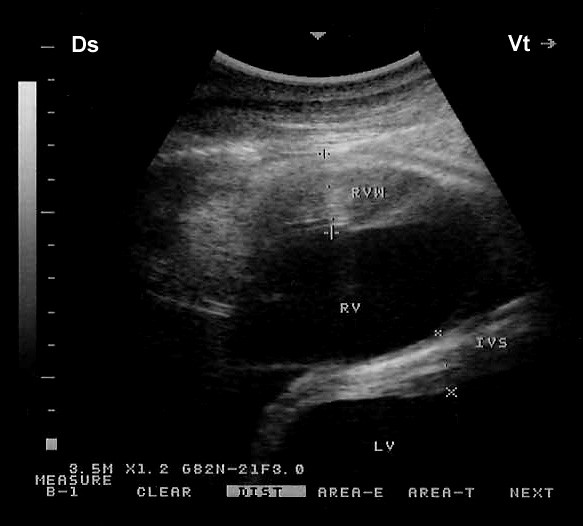
**Right short-axis view of the cardiac ventricles. Both ventricles are seen in a transverse section.** Ds, dorsal; Vt, ventral; RVW, right ventricular wall; RV, right ventricle; IVS, interventricular septum; LV, left ventricle.

**Figure 6:**
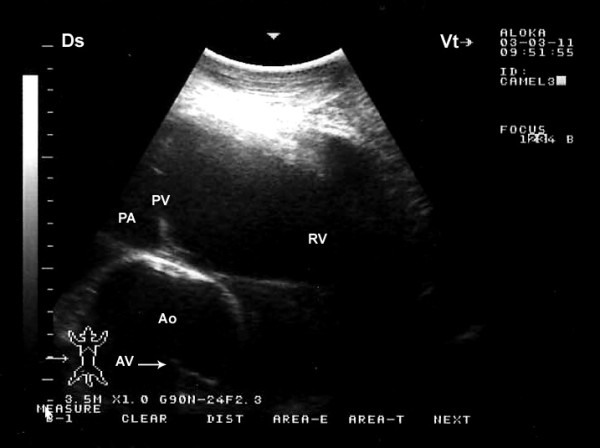
**Right parasternal cranial long-axis view of the right ventricular outflow tract on the third intercostal space.** Ds, dorsal; Vt, ventral; RV, right ventricle; PA, pulmonary artery; PV, pulmonary valve; Ao, aorta; AV, aortic valve.

Placing the probe slightly more cranially in the 4^th^ ICS with the transducer rotated cranially, the caudal long-axis view of the LVOT (the left ventricle, left atria, aortic valve, and the aortic root) were imaged in 17 camels (Figure 3). From this position, the right and left ventricles and interventricular septum (IVS) were visible in all camels, the right atrium in 19, the left atrium in 18, the tricuspid valve in 20. In 7 camels, a hybrid view between a “four-chamber” and “LVOT view” was imaged from the same position (Figure 4). A slight clockwise rotation in the 4^th^ ICS, the short-axis view of the cardiac ventricles was obtained (Figure 5). In 19 of the camels, the right ventricle, interventricular septum and left ventricle were visible, while in the other 3 were not. Placement of the transducer in the 3^rd^ ICS allowed the visualisation of the RVOT in which the right ventricle, the pulmonary valve, pulmonary artery, aorta and aortic valve were imaged in 19 camels (Figure 6).

### Left parasternal ultrasonograms

**Figure 7:**
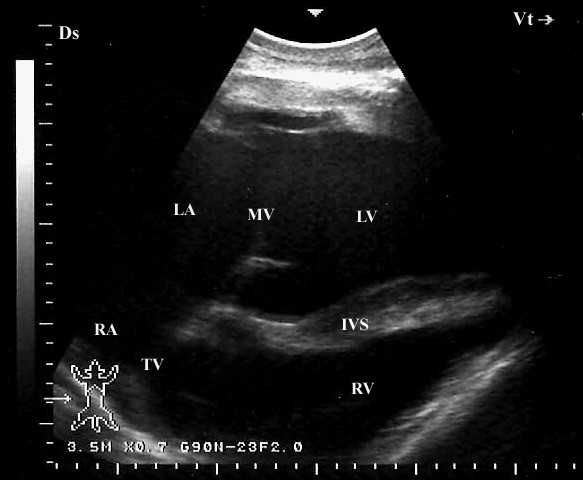
**Left parasternal caudal long-axis view of the heart. In this view, the four cardiac chambers are observed as well as the atrioventricular valves.** Ds, dorsal; Vt, ventral; LA, left atrium; LV, left ventricle; MV, mitral valve; RA, right atrium; RV, right ventricle; TV, tricuspid valve; IVS, interventricular septum.

**Figure 8:**
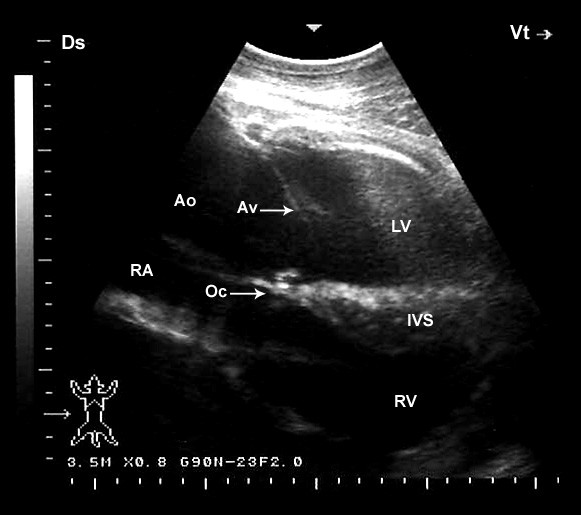
**Left parasternal caudal long-axis view of the left ventricular outflow tract. The left ventricle and aorta are observed.** The transversa view of the aortic valve is recognized as a thin echoic line. Ds, dorsal; Vt, ventral; RV, right ventricle; LV, left ventricle; RA, right atrium; IVS, interventricular septum, Ao, aorta; AV, aortic valve; Oc, *ossa chordis*.

**Figure 9:**
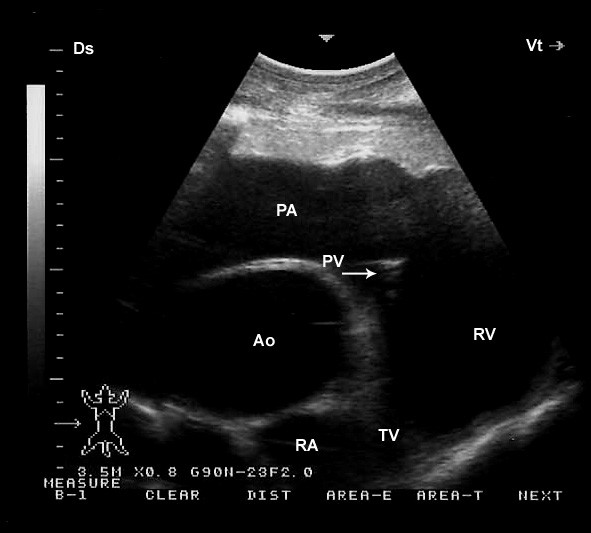
**Left parasternal cranial long-axis view of the right ventricular outflow tract.** The left ventricle and aorta are observed. The transversa view of the aortic valve is recognized as a thin echoic line. Ds, dorsal; Vt, ventral; RV, right ventricle; RA, right atrium; TV, tricuspid valve; Ao, aorta; PA, pulmonary artery; PV, pulmonary valve.

When the probe was placed longitudinally in the 5^th^ or 4^th^ ICS and directed slightly caudodorsally, a view of the ventricles, atria, and the atrioventricular valves was obtained (Figure 7). In this position, the left ventricle and the mitral valve were visible in 20 camels, the interventricular septum in all camels, the right ventricle in 19 and the tricuspid valve in 18 camels. The LVOT was imaged in the 4^th^ ICS and the probe is turned slightly more cranially and rotated slightly counter clockwise (Figure 8). In this position the right ventricle, tricuspid valve and the right atrium were imaged in 16 camels. The *ossa chordis* was also visible in the same position as a sub-aortic hyperechoic thin shadowing area in 15 of the 22 camels (68%). The RVOT was seen from the 3^rd^ intercostal space (Figure 9). In this position, the right ventricle and the tricuspid valve were visible in 14 camels and the right atrium in 9. An oblique section of the aorta was visible in 18 camels. The pulmonary artery and valve were visible in 21 and 19 camels, respectively.

The minimum, maximum, mean values, standard deviations and coefficient of variation for the measured variables are summarised in Table 1.

**Table 1:** Internal echocardiographic measurements in healthy adult camels (*n*=22)

**Variable**	**Min**	**Max**	**Mean**	**SD**	**CV**
RVd (cm)	4.3	8.0	5.3	1.2	22%
RVs (cm)	4.2	4.6	4.1	0.4	10%
LVd (cm)	9.9	15.5	11.8	1.6	14%
LVs (cm)	7.8	9.4	8.2	0.6	10%
RAd (cm)	5.0	6.5	5.9	0.5	10%
RAs (cm)	3.0	4.5	3.9	0.4	10%
LAd (cm)	6.8	8.8	7.6	0.6	10%
LAs (cm)	4.7	6.4	5.6	0.5	10%
RVWd (cm)	1.5	2.4	1.8	0.2	10%
RVWs (cm)	1.2	2.0	1.5	0.2	13%
IVSd (cm)	1.7	3.0	2.1	0.3	14%
IVSs (cm)	1.2	3.9	2.8	0.7	30%
LVWd (cm)	1.8	3.4	2.8	0.4	14%
LVWs (cm)	1.1	2.4	1.9	0.4	21%
AOd(cm)	6.0	8.2	7.0	0.8	11%
PAd (cm)	6.0	9.7	8.1	1.2	15%
TVDs (cm)	2.6	6.8	4.1	1.1	27%
MVDs (cm)	4.3	7.8	6.2	1.0	16%

N, number of the camels; Min, minimum value; Max, maximum value; SD, standard deviation;CV, coefficient of variation; RV, right ventricular diameter; LV, left ventricular diameter; RA right atrium diameter; LA, left atrium diameter; RVW, right ventricular wall thickness; IVS, interventricularseptal thickness; LVW, left ventricular wall thickness; AO, aortic diameter; PA, pulmonary artery diameter; TVD, tricuspid valve diameter; MVD, mitral valve diameter; d, diastole; s, systole.

## Discussion

Echocardiography allows investigation of the morphology and function of cardiac structures and measurement of cardiac dimensions. In cattle, dogs and horses, measurement of cardiac chamber dimensions is considered one of the most important tools for assessing heart disease severity and prognosis [8,9,11,12,20]. Recently, normal echocardiographic parameters of 1-year-old camels were reported [21]. In the adult camels, however the lack of data regarding normal echocardiographic patterns and internal cardiac dimensions hinders the progress in the diagnosis of camel cardiovascular diseases. This study is the first defining that cardiac images and measurements of internal cardiac structures can be obtained from adult camels. Knowledge of the normal appearance and cardiac dimensions should improve identification, quantification and assessment of cardiac disease and may allow an earlier diagnosis and more prompt intervention when facing to abnormal echocardiographic findings.

To achieve some of the echocardiographic images in cattle, physical strength is classically required [17]. The thoracic limbs should be moved cranially or gently abducted to facilitate better contact between the probe and the ICS [9]. As reported in cattle [8], most of the camels in this study were unwilling to move their forelimbs cranially, but were much more tolerant of the limb being abducted. In camels, ultrasonographic examination at standing position is not always without danger for the operator [1]. Therefore ultrasonographic examination of the camel is always performed at sitting position in a rather than standing position both in health [22-25] and in diseased status [26,27] requiring in addition to light sedation to slightly sedate the nervous dromedary camels. In the present study, examination of the camels in the recumbent position constituted a further challenge for the examiners. Images acquired from the 3^rd^ ICS on both sides required an assistance of 2 helpers. Therefore, the most difficult views to obtain were the short-axis views.

As reported in cows [8], the short-axis views were technically challenging due to difficulties in obtaining symmetrical images and poor visualisation due to the hyperechogenicity of the pleural surface. In addition, narrowing of the thoracic intercostal spaces, large size of the probe used and negative impact of the costal bone on the images produced were additional challenges. It has been reported previously in both horses and cattle that intercostal space width was a limiting factor in the quality of the images obtained [17,28]. In cattle, attenuation of the ultrasound beam by reflection and absorption causes significant deterioration of the image in animals with thick thoracic walls [17]. However, we did not encounter this problem in this study as camels were selected with a medium BCS.

The atria, ventricles and heart valves could be imaged in the majority of the camels. Exceptions included camels in which the lungs extended far cranially and obscured the atria. The pulmonary valve was imaged from the right and left 3^rd^ ICS, but was best imaged from the left 3^rd^ ICS. This result agrees with the findings in cattle visualizing the pulmonary valve in the 3^rd^ left ICS [17]. The aortic valve could be best imaged from the right 4^th^ ICS. It could be also imaged in the 3^rd^ ICS. This result agrees also with the same study in cows, but they visualized the aortic valve only in the 4^th^ ICS [17].

During echocardiography, a phased array probe is preferred, if available. However, a large sectorial or even linear probe is generally sufficient. In the present study, although the echocardiographic examination was carried out using a 3.5-MHz sectorial transducer, which was the only available probe, it was effective in performing all the scanning views of the heart. In a study reported recently in cattle by our group, using a 3.5-MHz linear transducer was effective in detecting pulmonic, tricuspid and mitral valve vegetation [10]. In another study in buffaloes reported also by our group, using a 3.5-MHz sectorial transducer was effective in determining pericardial effusions, fibrinous pericarditis, suppurative pericarditis, tricuspid valve vegetation, mitral valve vegetation and pulmonary valve vegetation [29]. In this study, some differences in probe placement were noted compared to other ruminant studies [8-10]. The right parasternal long-axis four-chamber view was obtained with the probe placed in the 5^th^ ICS. In addition, the LVOT on the right 4^th^ ICS was orientated differently from other bovine studies [8,9,17]. The LVOT in most of the camels could be visualized together with the four-chamber view at the right 4^th^ ICS. Internal cardiac measurements have been shown to be extremely valuable in dogs, cattle and horses [9,12,30]. It assumes normal ventricular morphology, afterload, preload and ventricular contractility [31].

This study has its own limitations. One of these limitations was the potential influence of sedation on some of the echocardiographic measurements. To decrease such possibility, we used only mild sedation by xylazine (0.02 mg/kg IV instead of the regular sedation dose of 0.1 mg/kg). Although the clinical significance of the α_2_ antagonist, xylazine may be minimal, its potential effects should be taken into consideration when echocardiographic variables are interpreted in clinical cases. Another limitation of this study was the using of a machine with a maximum imaging depth of 22 cm, low sector width and frame rate. To overcome these limitations, M-mode recordings, which provides a considerably higher frame rate and is routinely used for measurement of linear LV dimensions, was used together with 2-D recordings. A third limitation of this study was the unavailability to use a simultaneous ECG to facilitate the detection of end-diastole. We can reasonably say that this study can be easily translated in the field since most of clinics carrying out large animal echocardiography don’t have specific probes. Further studies are therefore still required for better characterising the cardiovascular function of healthy camel especially focusing on their stroke volume and cardiac output depending on their physical state.

## Conclusions

The data presented in this paper demonstrates that it is possible to obtain good quality echocardiograms in adult camels and provide normal cardiac dimensions to compare against diseased animals. This study is thus relevant for the diagnosis of early manifestations of cardiac disease in adult camels. The technique is relatively straightforward but does require some physical strength. Application of the normal ranges generated in this study has the potential to confirm or deny the presence of cardiac diseases in camel patients with cardiovascular disease. In addition, echocardiographic measurements in the healthy camels may be used as prognostic values in sick camels with heart diseases. In this regard, echocardiographic findings should be compared with the normal reference ranges described in this study.

## Authors’ contributions

MT and SB initiated and planned the study. MT, FA and AA carried out the ultrasonographic and postmortem examinations. MT wrote the manuscript and made the figures. SB read and revised the manuscript. All authors have read and approved the manuscript.

## Authors' information

MT: DVM, MSc, PhD, Associate professor, Department of Veterinary Medicine, College of Agriculture and Veterinary Medicine, Qassim University, Saudi Arabia. Permanent address: Department of Animal Medicine, Faculty of Veterinary Medicine, Zagazig University, Egypt. FA: DVM, MSc, PhD, Professor, Department of Veterinary Medicine, College of Agriculture and Veterinary Medicine, Qassim University, Saudi Arabia. AA: DVM, MSc, PhD, Professor, Department of Veterinary Medicine, College of Agriculture and Veterinary Medicine, Qassim University, Saudi Arabia. SB: Dr Vét, DÉS, MSc, DACVIM, Associate professor, Clinique ambulatoire bovine/Bovine ambulatory clinic, Faculté de médecine Vétérinaire, Université de Montréal, CP 5000, St-Hyacinthe, J2S 7 C6, Québec, Canada, Tel/Phone 450 773 8521 ext 8675, Fax 450 778 8120.
